# Conservation of a conformational switch in RadA recombinase from *Methanococcus maripaludis*

**DOI:** 10.1107/S0907444909011871

**Published:** 2009-05-15

**Authors:** Yang Li, Yujiong He, Yu Luo

**Affiliations:** a Department of Biochemistry, University of Saskatchewan, A3 Health Sciences Building, 107 Wiggins Road, Saskatoon, Saskatchewan S7N 5E5, Canada

**Keywords:** RadA, Rad51, RecA, recombinases, homologous recombination, DNA strand exchange, ATPases

## Abstract

Archaeal RadAs are close homologues of eukaryal Rad51s (∼40% sequence identity). These recombinases promote ATP hydrolysis and a hallmark strand-exchange reaction between homologous single-stranded and double-stranded DNA sub­strates. Pairing of the 3′-overhangs located at the damaged DNA with a homologous double-stranded DNA enables the re-synthesis of the damaged region using the homologous DNA as the template. In recent studies, conformational changes in the DNA-interacting regions of *Methanococcus voltae* RadA have been correlated with the presence of activity-stimulating potassium or calcium ions in the ATPase centre. The series of crystal structures of *M. maripaludis* RadA presented here further suggest the conservation of an allosteric switch in the ATPase centre which controls the conformational status of DNA-interacting loops. Structural comparison with the distant *Escherichia coli* RecA homologue supports the notion that the conserved Lys248 and Lys250 residues in RecA play a role similar to that of cations in RadA. The conservation of a cationic bridge between the DNA-interacting L2 region and the terminal phosphate of ATP, together with the apparent stability of the nucleoprotein filament, suggests a gap-displacement model which may explain the advantage of ATP hydrolysis for DNA-strand exchange.

## Abstract 2

Structural conservation in the ATPase centers of RadA, Rad51 and RecA recombinases suggests conformational switching between high and low-affinity states for DNA in concert with cycles ATP hydrolysis. Such iteration would be advantageous for DNA strand exchange by optimizing the pairing between single-stranded and double-stranded DNA substrates.

## 1. Introduction

The bacterial RecA (Clark & Margulies, 1965 ▶), archaeal RadA (Sandler *et al.*, 1996 ▶) and eukaryal Rad51 (Shinohara *et al.*, 1992 ▶) and DMC1 (Bishop *et al.*, 1992 ▶) proteins, which belong to a recombinase superfamily (Seitz & Kowalczykow­ski, 2000 ▶), facilitate a central DNA strand-exchange process between a single-stranded DNA (ssDNA) and a homologous double-stranded DNA (dsDNA) in homologous recombination. This recombinase-facilitated process is essentially identical to the denaturing and annealing steps in a PCR reaction except that no temperature manipulation is required. Homologous recombination appears to be essential by playing a pivotal role in the repair of double-stranded DNA breaks and the restarting of stalled replication forks (Cox, 1998 ▶; Cox *et al.*, 2000 ▶; Courcelle *et al.*, 2001 ▶; Lusetti & Cox, 2002 ▶; Kowalczykowski, 2000 ▶). Despite large differences between the bacterial and non­bacterial recombinases in their primary structures (less than 30% sequence identity), electron-microscopic and crystallo­graphic results have revealed strikingly similar ‘active’ recombinase assemblies (VanLoock *et al.*, 2003 ▶; Conway *et al.*, 2004 ▶; Wu *et al.*, 2004 ▶; Chen *et al.*, 2008 ▶; Sheridan *et al.*, 2008 ▶). The structures of EcRecA^1^ in complex with a series of DNA molecules have shed light on the exact mechanism of homologous DNA-strand exchange (Chen *et al.*, 2008 ▶). These right-handed helical recombinase assemblies are classic allosteric systems equipped with two functional sites: one located at the subunit interface for binding and hydrolyzing ATP and the other near the filament axis for binding DNA and promoting strand exchange. In the very first crystal structure of EcRecA in complex with ADP, the DNA-binding regions L1 and L2 are disordered in an apparently inactive form (Story *et al.*, 1992 ▶). The L1 and L2 regions of other crystallized inactive filaments of bacterial RecAs are either disordered (Datta *et al.*, 2000 ▶; Rajan & Bell, 2004 ▶; Xing & Bell, 2004*a* ▶,*b* ▶) or observed in an ensemble of conformations (Datta, Ganesh *et al.*, 2003 ▶; Datta, Krishna *et al.*, 2003 ▶; Krishna *et al.*, 2007 ▶). It is increasingly clear that the conformations of these loops are controlled by allosteric effectors such as the nucleotide cofactor (Datta, Ganesh *et al.*, 2003 ▶; Datta, Krishna *et al.*, 2003 ▶; Krishna *et al.*, 2007 ▶). Strikingly, these loops become highly ordered around the bound DNA in the recently crystallized active form of EcRecA in complex with ADP–AlF_4_ and DNA (Chen *et al.*, 2008 ▶). Similar conformational changes of the DNA-binding regions have also been observed in crystal structures of MvRadA (Wu *et al.*, 2005 ▶; Qian, He, Ma *et al.*, 2006 ▶). The cation-dependence of RadA enabled us to propose the hypothesis of the existence of a conformational switch in the ATPase centre (Wu *et al.*, 2005 ▶). Since such cation-dependence appears to be ubiquitous in the closely related group of archaeal RadA (Wu *et al.*, 2005 ▶; Qian, He, Wu *et al.*, 2006 ▶; Qian, He, Ma *et al.*, 2006 ▶), eukaryal Rad51 (Rice *et al.*, 2001 ▶; Liu *et al.*, 2004 ▶; Sehorn *et al.*, 2004 ▶; Shim *et al.*, 2006 ▶; Bugreev & Mazin, 2004 ▶) and DMC1 (Bugreev *et al.*, 2005 ▶; Lee *et al.*, 2005 ▶) proteins, it should be possible to observe con­certed conformational changes in another homologue. We set out to study conformational changes in several RadA recombinases. MmRadA, a close homologue of MvRadA (∼81% sequence identity), turned out to be amenable to crystallization. Similar to MvRadA, crystallized MmRadA has been observed in two sets of conformations correlated with the presence or absence of a stimulating concentration of ammonium or potassium ions. Structural comparison with its distant EcRecA homologue supports the notion that the two invariant lysine residues (residues 248 and 250 in EcRecA) in bacterial RecAs play a role similar to that of monovalent cations in RadA (Wu *et al.*, 2005 ▶; Qian, He, Wu *et al.*, 2006 ▶).

## 2. Materials and methods

### 2.1. Cloning, protein preparation and crystallization

The open reading frame of RadA from *M. maripaludis* was inserted between the *Nco*I and *Xho*I sites of pET28a (Nova­gen). The resulting plasmid was verified by DNA sequencing using T7 promoter and terminator primers. The recombinant MmRadA was overexpressed in BL21 Rosetta2 (DE3) cells (Novagen) and purified as reported for MvRadA (Wu *et al.*, 2004 ▶; Qian, He, Wu *et al.*, 2006 ▶). In brief, the purification procedure involved steps of polymin P (Sigma) precipitation and high-salt extraction and three chromatographic steps using heparin (Amersham Biosciences), hydroxyapatite (Bio-Rad) and DE52 anion-exchange (Whatman) columns. The purified MvRadA protein was concentrated to ∼30 mg ml^−1^ by ultrafiltration.

### 2.2. ATPase assay

A solution containing 0.033%(*w*/*v*) Malachite Green, 1.3%(*w*/*v*) ammonium molybdate and 1.0 *M* HCl was used to monitor the release of inorganic phosphate (Itaya & Ui, 1966 ▶) by ATP hydrolysis. The absorbance at 620 nm was recorded for quantification. The reaction solutions for the ssDNA-dependent ATPase assay contained 3 µ*M* RadA, 18 µ*M* ssDNA (in nucleotides), 5 m*M* ATP, 0.05 *M* Tris–HEPES buffer pH 7.4, 100 m*M* of a specified monovalent salt, 10 m*M* MgCl_2_ and 0.1%(*v*/*v*) 2-mercaptoethanol. The 36-nucleotide oligonucleotide poly-(dT)_36_ (Integrated DNA Technologies) was used as the ssDNA substrate. The conditions for the high-salt-stimulated ATPase assay were similar except for the presence of a 1.0 *M* concentration of a specified monovalent salt as a substitute for the ssDNA.

### 2.3. Strand-exchange assay using synthetic oligonucleotides

The DNA substrates were modified based on a published study (Mazin *et al.*, 2000 ▶). Three oligonucleotides (#FAM43, 43 nucleotides, FAM-TTTTG CGGAT GGCTT AGAGC TTAAT TGCTG AATCT GGTGC TGT; #45A, 36 nucleotides, ACAGC ACCAG ATTCA GCAAT TAAGC TCTAA GCCATG; #55A, 36 nucleotides, GATGG CTTAG AGCTT AATTG CTGAA TCTGG TGCTGT) were obtained from Integrated DNA Technologies. Equal molarities of complementary oligonucleotides #45A and #55A were heated at 368 K for 5 min and then slowly cooled to 294 K to generate the 36 bp dsDNA substrate. The strand-exchange solution was composed of 5 m*M* ATP or an analogous nucleotide, 10 m*M* MgCl_2_, 100 m*M* of a specified monovalent salt, 50 m*M* HEPES–Tris buffer pH 7.4, 15 µ*M* MvRadA, 0.1%(*v*/*v*) 2-­mercaptoethanol and 1 µ*M* oligonucleotides. The 43-nucleotide 5′-fluorescein-labelled ssDNA substrate (oligo­nucleotide #FAM43) was pre-incubated at 310 K with MvRadA for 1 min before adding the 36 bp dsDNA substrate. The reaction was stopped after 30 min by adding EDTA to a concentration of 20 m*M* and trypsin to a concentration of 1 µg µl^−1^. After 10 min of proteolysis, 10 µl sample was mixed with 5 µl loading buffer composed of 30% glycerol and 0.1% Bromophenol Blue, loaded onto a 17% SDS–polyacrylamide vertical gel and developed for 1 h at 100 V. The fluorescent emission by the intrinsic fluorescein was recorded using a Kodak GelLogic 200 system.

### 2.4. Crystallization of MmRadA and diffraction data collection

Hexagonal MmRadA crystals (space group *P*6_1_) were grown by the hanging-drop vapour-diffusion method and grew to maximum dimensions of 0.1 × 0.1 × 0.3 mm. The protein sample contained ∼1 m*M* concentrated RadA and 2 m*M* AMPPNP. The reservoir solutions contained 6–8% polyethylene glycol 3350, 50 m*M* MgCl_2_, 50 m*M* Tris–HCl buffer pH 7.9 and 0.1–0.4 *M* of a monovalent salt (NaCl, KCl, RbCl or NH_4_Cl). The crystals were transferred into a stabilizing solution composed of the reservoir solution supplemented with 28%(*w*/*v*) sucrose, looped out of the solution and frozen in a nitrogen cryostream at 100 K. The diffraction data sets were first collected using an in-house Bruker Proteum system at a wavelength of 1.54 Å and subsequently using synchrotron radiation at a wavelength of 0.97 Å. The synchrotron data sets were processed using the *XDS* program (Kabsch, 1993 ▶). The statistics of the diffraction data are listed in Table 1 ▶.

### 2.5. Structural determination and refinement

The previously solved MvRadA model (PDB code 1t4g) was used as the starting model. Rigid-body refinement using low-resolution data (20–6.0 Å) was initially employed. The models were then iteratively rebuilt using *XtalView* (McRee, 1999 ▶) and refined using *CNS* (Brünger *et al.*, 1998 ▶). Statistics of the refinement and model geometry are also given in Table 1 ▶. The molecular figures were generated using *MOL­SCRIPT* (Kraulis, 1991 ▶) and rendered using *RASTER*3*D* (Bacon & Anderson, 1988 ▶). The coordinates and structure factors have been deposited in the Protein Data Bank (PDB codes 3etl, 3ew9 and 3ewa).

## 3. Results

### 3.1. DNA-dependent ATPase activity is selective towards monovalent cations

**Figure 1:**
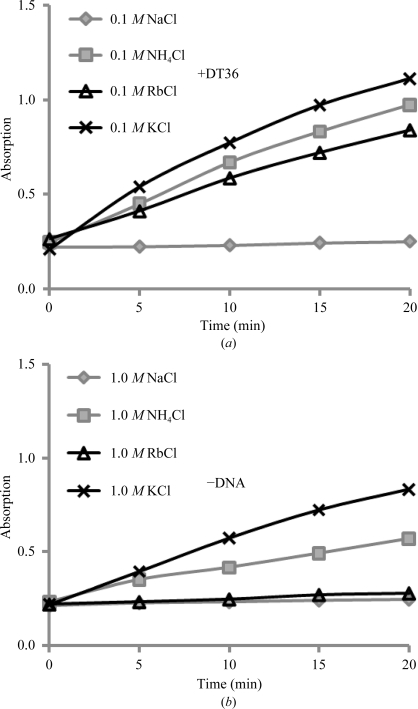
Monovalent cation-dependence of ATP hydrolysis catalyzed by MmRadA. The reaction solutions contained 5 m*M* ATP, a specified amount of a monovalent salt, 10 m*M* MgCl_2_ and 50 m*M* HEPES–Tris buffer pH 7.4. The solutions also contained 3 µ*M* MmRadA. (*a*) Time courses of phosphate release in the presence of 100 m*M* of a specified monovalent salt and 1 µ*M* poly-(dT)_36_. (*b*) Time courses in the presence of 1.0 *M* of a specified monovalent salt. DNA was absent.

As reported previously, potassium is the only commonly used monovalent cation which stimulates the ATPase activity of MvRadA (Wu *et al.*, 2005 ▶). We first studied the ATPase activity of the MmRadA homologue in the presence of an ssDNA [poly-(dT)_36_] and 100 m*M* of a monovalent cation (Na^+^, NH_4_^+^, Rb^+^ or K^+^; Fig. 1 ▶ *a*). An MmRadA:ssDNA ratio of 1:6 (six nucleotides per protein) was used in this assay. As expected from its high level of similarity to MvRadA (∼81% sequence identity), the *M. maripaludis* protein also actively catalyzed ATP hydrolysis in the presence of potassium but not of sodium. However, MmRadA appears to be less selective than MvRadA towards monovalent cations. In the presence of the similarly sized ammonium or rubidium ions, the ATPase activities were similar (over 60%) to that in the presence of potassium.

### 3.2. High-salt-stimulated ATPase activity is selective towards monovalent cations

Many bacterial RecA proteins, as well as eukaryal Rad51 and archaeal RadA proteins, are known to become ATPase-active in the presence of high salt as a substitute for ssDNA (Pugh & Cox, 1988 ▶; Tombline & Fishel, 2002 ▶; Liu *et al.*, 2004 ▶; Wu *et al.*, 2005 ▶). As expected, MmRadA shares this property in the presence of 1.0 *M* KCl or NH_4_Cl (Fig. 1 ▶ *b*). Although RbCl stimulated the ssDNA-dependent ATPase activity of MmRadA, 1.0 *M* RbCl did not noticeably stimulate the ATPase activity of MmRadA in the absence of DNA.

### 3.3. DNA-strand exchange activity is selective towards monovalent cations

**Figure 2:**
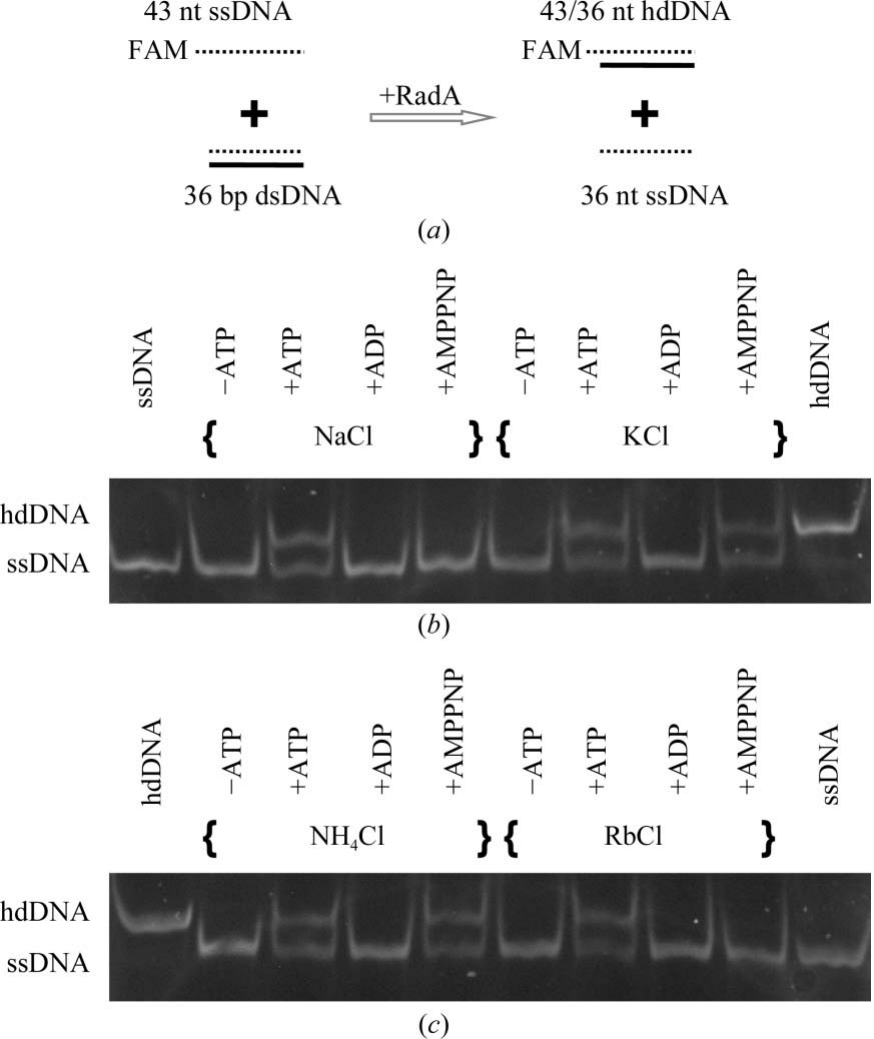
Monovalent cation-dependence of the DNA strand-exchange activity of MmRadA. The strand reaction scheme is shown in (*a*). The reaction solutions contained 2 m*M* of a specified nucleotide, 100 m*M* of a specified monovalent salt, 10 m*M* MgCl_2_ and 50 m*M* HEPES–Tris buffer pH 7.4. The solutions also contained 15 µ*M* MmRadA and 1 µ*M* each of ssDNA (FAM-labelled) and dsDNA (unlabelled) substrates. Strand-exchange activity was indicated by the formation of the slowest migrating heteroduplex DNA species (FAM-labelled hdDNA). The intrinsic fluorescence from the FAM label was recorded as shown in (*b*) and (*c*).

In the strand-exchange assay (Fig. 2 ▶), an MmRadA:ssDNA ratio of 1:3 (three nucleotides per protein) was used, which conforms to the known stoichiometry for such recombinases. In the presence of ATP, the *M. maripaludis* protein was active for all four monovalent cations tested. In the presence of AMPPNP, however, it was active with only two (K^+^ and NH_4_^+^) of the four cations.

### 3.4. The crystal structure of an ATPase-active conformation of MmRadA

**Figure 3:**
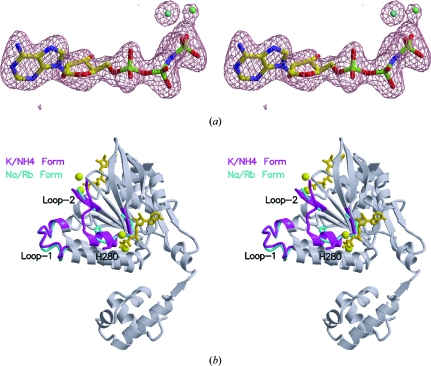
Crystal structure of MmRadA in complex with AMPPNP in stereo. (*a*) OMIT (*F*_o_ − *F*_c_) difference Fourier map at 2.0 Å resolution. The AMPPNP molecule is shown as a stick model. The possible locations of two ammonium ions are shown as cyan spheres. (*b*) Two conformational forms of MmRadA. Two AMPPNP molecules and the side chains of His280 are shown as stick models. The protein is shown in ribbon representation. The DNA-interacting loops are highlighted in cyan for the recurrent inactive form observed in NaCl, RbCl or a low concentration (<0.3 *M*) of KCl or NH_4_Cl. They are highlighted in magenta for the ATPase-active form observed in 0.4 *M* KCl or NH_4_Cl.

Since KCl and NH_4_Cl were observed to stimulate the ATPase activity of MmRadA in the absence of ssDNA as well as the strand-exchange activity of MmRadA in the presence of AMPPNP, we reasoned that both salts could serve as stabilizing reagents for MmRadA to remain predominantly in its ATPase-active conformation. In the presence of 0.4 *M* of either salt, MmRadA was indeed crystallized in a largely ordered conformation (except for seven residues from Pro262 to Val268) similar to the ATPase-active conformation seen in MvRadA (Wu *et al.*, 2005 ▶; Table 1 ▶). The two MmRadA structures are essentially identical except for the higher resolution data obtained for the crystal of MmRadA in the presence of 0.4 *M* NH_4_Cl. All crystals reported in this study were grown under similar conditions apart from the concentration and identity of the monovalent salt. The crystals belong to space group *P*6_1_, with similar helical pitches (107.1–108.5 Å) coinciding with the crystallographic *c* axis (Table 1 ▶). Although this packing scheme appears to be similar to that reported for MvRadA (104–107 Å pitch), the mode of inter-filament interaction is different. In MvRadA crystals, the crystal packing is stabilized by a repeated mode of cation-bridged inter-filament interaction between an N-terminal turn (Thr6–Val11) and Glu164. Three carbonyl groups (Thr6, Leu8 and Val11) and the side chain of Glu164 coordinate the bridging Na^+^ or K^+^. In MmRadA crystals, the role of the N-­terminal turn in inter-filament packing is similar. The cation-bridged interaction, however, is replaced by direct hydrogen bonds between the three carbonyl groups of the N-­terminal turn and the side chain of Lys318. The helical pitch ranges from 107.1 to 107.2 Å in this ATPase-active form obtained in the presence of 0.4 *M* KCl or NH_4_Cl. The non­hydrolyzable ATP analogue AMPPNP is buried between the MmRadA monomers as expected. An OMIT difference electron-density map of the crystal in the presence of 0.4 *M* NH_4_Cl revealed two solvent molecules near the γ-phosphate of the ATP analogue (Fig. 3 ▶ *a*). These are probably ammonium ions, since both are located at positions corresponding to the potassium ions in the structure with 0.4 *M* KCl (map not shown). The twin ions are likely to provide polarization of the γ-­phosphate in addition to the electron-withdrawing effects by the well known P-loop and magnesium ion (Saraste *et al.*, 1990 ▶), therefore suggesting compatibility of this conformation with requirement for efficient ATP hydrolysis. As seen in the ATPase-active conformation of MvRadA, the MmRadA structure is also highly ordered in its DNA-binding L1 and L2 region (in magenta; Fig. 3 ▶ *b*). Noticeably, an eight-residue helix (residues Gly275–Ala282) analogous to helix *G* of EcRecA (Story *et al.*, 1992 ▶) is ordered in the L2 region. Two K^+^/NH_4_^+^ ions (yellow spheres; Fig. 3 ▶ *b*) form bridges between the γ-­phosphate and the backbone carbonyl moieties at the C-­terminus of the eight-residue helix. In addition, the side chain of His280 makes a direct hydrogen bond to the γ-­phosphate. The root-mean-square deviation between the ATPase-active conformations of MvRadA (PDB code 2fpm) and MmRadA is 0.58 Å for 311 superimposed C^α^ atoms. The positions of the twin potassium/ammonium ions are essentially identical.

### 3.5. The crystal structure of MmRadA in an inactive conformation

The second set of MmRadA–AMPPNP structures were determined using crystals in the presence of 0.1–0.4 *M* NaCl or RbCl or 0.1–0.3 *M* KCl or NH_4_Cl. These conditions were selected because they did not appear to provide adequate cationic stabilization to trap the protein in its ATPase-active conformation. Indeed, less ordered structures that were essentially identical to each other were observed. The highest resolution data are listed in Table 1 ▶. In these structures, no plausible ions were seen even for Rb^+^, which is an electron-rich anomalous scatterer. As such, the catalyzing effect of the monovalent cations seen in the ATPase-active conformation is lost in this second set of MmRadA structures. Compared with the ATPase-active filaments of MmRadA, the filament pitch is slightly longer (108.5 Å). Since very similar filament pitches were observed for multiple data sets under similar ionic conditions (within 0.2 Å), the difference in helical pitches appeared to be significant. Although the DNA-binding L1 region remains ordered, the L2 region becomes largely dis­ordered (shown in cyan in Fig. 3 ▶ *b*). There was no electron density for 19 residues from Ala260 to Val278. The root-mean-square deviation between this second conformation of MmRadA and the previously reported ATPase-inactive con­formation of MvRadA (PDB code 1t4g) is 0.64 Å for 299 superimposed C^α^ atoms, thus confirming the reoccurrence of a similar inactive conformation in MmRadA.

## 4. Discussion

### 4.1. Comparison with electron-microscopy-derived filament parameters

MmRadA is the second strand-exchange protein of the RadA/Rad51/DMC1 group to have been crystallized in a form resembling the active filament. Despite its high level of sequence similarity to MvRadA, differences in crystal packing suggest that stabilizing interactions in the crystal lattice did not dictate the conformations observed. As in MvRadA, two sets of conformations have been observed, each resembling the corresponding set in MvRadA. We have con­ducted soaking experiments with MmRadA crystals. It appears that the conformation is solely determined by the chemical composition of the soaking solution and not by the initial crystallizing solution. Therefore, the ATPase centre and DNA-binding loops of MmRadA are likely to remain mobile in the crystal lattice, which resembles the state in solution. The values of helical pitch and number of subunits per turn in the recently determined structure of active EcRecA filament in complex with DNA (∼94.0 ± 1.5 Å and 6.16 ± 0.03 subunits per turn; Chen *et al.*, 2008 ▶) are in close agreement with those derived from electron microscopy (96 Å pitch and 6.2 subunits per turn on average; Sheridan *et al.*, 2008 ▶). On the other hand, electron microscopy revealed that active filaments of eukaryal Rad51 and DMC1 proteins have ∼6.5 subunits per turn and average pitches ranging from 101 to 103 Å (Sheridan *et al.*, 2008 ▶). As a close homologue of the eukaryal strand-exchange proteins, MvRadA filaments formed in the presence of ADP–AlF_4_ have a slightly shorter average pitch of ∼99 Å and ∼6.25 subunits per turn (Galkin *et al.*, 2006 ▶). The crystal structures of the high-salt-stabilized ATPase-active form of MvRadA have an average pitch of ∼105 Å and are apparently overwound, with exactly six subunits per turn. The crystallized ATPase-active filaments of MmRadA, which are similarly overwound to MvRadA owing to crystal packing along the crystallo­graphic sixfold axis, have even longer pitches of around 107 Å. The longer pitch as well as the overwound nature is indicative of the differences between the active recombinase/DNA filament and the high-salt-stabilized ATPase-active forms of RadA recombinases, the latter of which appears to be more flexible and hence amenable to crystallization in space group *P*6_1_. Although the loop 2-anchoring helix *G* in the RadA structures appears to be similarly positioned to its counterpart in the EcRecA–DNA complex, as suggested by similar orientations of His280 and its equivalent Phe217 residue located at the C-terminus of this short helix, the overwound nature of the crystallized MvRadA and MmRadA filaments appeared to be incompatible with DNA binding. In fact, efforts to cocrystallize the two RadA proteins with DNA under similar conditions have been futile.

### 4.2. Structural comparison of the ATPase centres of MmRadA and EcRecA

**Figure 4:**
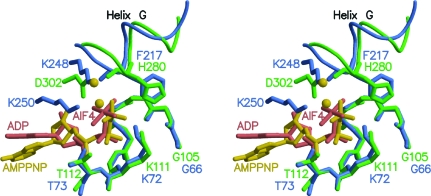
Superimposed ATPase centres of MmRadA and EcRecA in stereo. The conserved P-loops are superimposed. The P-loop (Gly105–Thr112), His280 and Asp302 of the ATPase-active form of MmRadA are shown as green stick models. The MmRadA-bound AMPPNP and monovalent cations are shown as yellow ball-and-stick models. The P-loop (Gly66–Thr73), Phe217, Lys248 and Lys250 of the active EcRecA structure are shown in blue.

The structure of the DNA-bound active form of EcRecA has recently been elucidated (Chen *et al.*, 2008 ▶). Since the RecA proteins are remote homologues of RadA with barely 30% sequence identity, it would be more meaningful to superimpose the conserved P-loops of EcRecA (PDB code 3cmx; residues 3066–3073) and MmRadA (residues 105–112). The resulting root-mean-square deviation between the 32 equivalent main-chain atoms of the P-loops is 0.30 Å. It is not surprising that the P-loop-wrapped triphosphate parts of the two structures are also placed in close vicinity (Fig. 4 ▶). The ribose and base counterparts, on the other hand, do not superimpose well. One noticeable difference in the ATP-interacting cap (equivalent to residues 302–308 of MvRadA) is that RecA proteins have two conserved lysyl residues (residues 248 and 250 in EcRecA), while RadA/Rad51/DMC1 proteins share an aspartyl residue (residue 302 in MmRadA and MvRadA). The tip of the side chain of the conserved Lys248 of EcRecA is placed ∼0.7 Å from the cation held in place by Asp302 of MmRadA. This lysyl side chain also forms a hydrogen bond to the carbonyl of Phe217 located at the C-­terminal end of helix *G*. In both RadA proteins the monovalent cation is coordinated by the carbonyl of His280 at the position equivalent to Phe217 of RecA. The other con­served lysyl residue in EcRecA, Lys250, is ∼2.7 Å from the second monovalent cation in the ATPase centre of RadA.

### 4.3. Conservation of a conformational switch in the ATPase centres

The obvious structural similarity between the ATPase centres of RadA and RecA proteins appears to suggest the existence of a conserved cationic bridge between the γ-­phosphate of ATP and the C-terminus of helix *G* (Fig. 4 ▶) which serves as a conformational switch controlled by the ATP-hydrolysis cycle. In the recently reported crystal structures of the active forms of MvRadA and EcRecA, this bridge appears to stabilize a highly ordered conformation of the DNA-interacting loop 2 that is compatible with a high-affinity state for DNA binding. ATP hydrolysis would almost certainly lead to the collapse of this stabilizing bridge and result in mobility in loop 2 and hence lower affinity for DNA. Such structural consequences have been observed in the MvRadA–ADP complex (Qian *et al.*, 2005 ▶), which is essentially similar to the inactive forms observed in the MvRadA–AMPPNP and MmRadA–AMPPNP complexes. Similar consequences could also occur for RecA proteins in the aftermath of ATP hydrolysis. Importantly, ATP hydrolysis does not necessarily lead to filament dissociation, although the inactive form appears to be adaptive to a wider range of helical pitches as seen in the much shorter pitched EcRecA (Story *et al.*, 1992 ▶; ∼82 Å pitch) and MvRadA devoid of its N-terminal domain (∼90 Å pitch; Galkin *et al.*, 2006 ▶).

### 4.4. Putative advantage of ATP hydrolysis for DNA strand exchange

The functional importance of ATP hydrolysis has always been an issue of debate for RecA-like strand-exchange proteins since the three-stranded exchange process can also be carried out in the absence of ATP hydrolysis (Kowalczykow­ski & Krupp, 1995 ▶; Sung & Stratton, 1996 ▶). Three models have been proposed, as reviewed by Cox (2007 ▶). Although these models can explain most features of RecA activity, inconsistencies with the known structural features of the recombinase assembly exist. The filament-dissociation model and RecA-redistribution model are largely based on hydrolysis-triggered RecA dissociation at the 5′-end or in the interior of the filament. Owing to massive stabilizing interactions between adjacent subunits within the filament, the dissociation of recombinase subunits should be minimal except for the filament ends. At the 5′-end of the nucleoprotein, filament dissociation would be blocked by recombinase-recruiting proteins such as bacterial RecFOR (Morimatsu & Kowalczykowski, 2003 ▶) and eukaryal BRCA2 (Yang *et al.*, 2005 ▶). Besides, direct observation of filament formation of RecA on dsDNA suggests that net growth at both ends is possible (Galletto *et al.*, 2006 ▶). Coupling of the ATP-hydrolytic cycle between adjacent RecA monomers is consistent with the observed filament-dissociation rate at the 5′-end and supports the third model of facilitated DNA rotation (Cox, 2003 ▶; Cox *et al.*, 2005 ▶). This model has provided an interpretation of how RecA clears unpaired segments by synchronized rotation of these segments. The available crystal structures of RecA-like proteins appear to suggest that ATP hydrolysis would lead to reduced affinity for DNA owing to conformational change in loop 2 and shortened pitch owing to rearrangement at the ATP-binding interface. However, there is no structural evidence to associate a motoring function with RecA-like proteins. The inconsistencies of the three models with the structural data of recombinases prompted us to rethink how ATP hydrolysis could facilitate the removal of such unpaired roadblocks without requiring the dissociation of recombinase subunits from the nucleoprotein filament or ATP-driven rotation of DNA.

**Figure 5:**
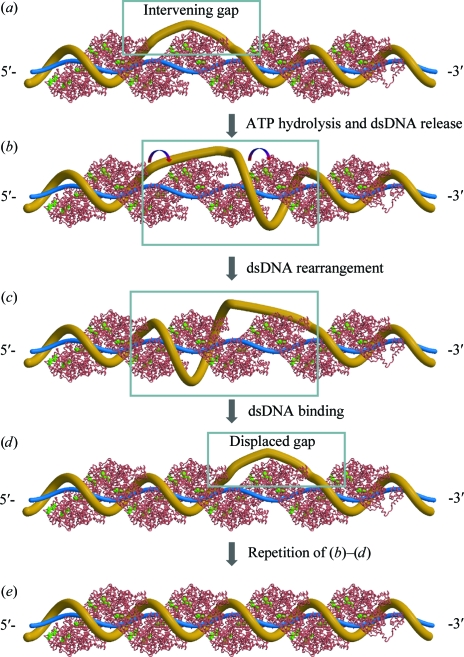
Hypothetical model of ATP hydrolysis-facilitated gap displacement. The crystallized protein filament is shown as a C^α^ trace in salmon with AMPPNP in green. Speculative models of the DNA substrates are shown as wires. Important segments are boxed. The 5′- and 3′-ends are based on the filament-initiating ssDNA (thinner wire in blue). The homologous dsDNA is shown as a thicker wire in yellow. The strand-exchange process progresses from the 5′-end to the 3′-end. (*a*) An intervening gap. Such gaps are likely to exist owing to simultaneous homologous pairing between the recombinase/ssDNA filament and dsDNA at multiple locations. The dsDNA in the gap region cannot become properly wound (∼19 bp per helical turn) around the nucleoprotein filament without unwinding its adjacent region(s). Despite the sequence homology, it serves as a topological roadblock of strand exchange between long DNA substrates. (*b*) ATP hydrolysis promotes the transient release of a dsDNA segment at the immediate 3′-flank of the gap. The transiently released dsDNA region is shown as an exaggerated wide helix. (*c*) Rearrangement in the transiently released dsDNA region and the adjacent gap takes place without changing the overall topology. The 5′-end of the gap region becomes properly wound, while the released dsDNA region becomes unwound. (*d*) The rearranged 5′-end of the gap becomes bound by the recombinase filament. As a result, the gap is displaced towards the 3′-end. (*e*) Repetition of steps (*b*)–(*d*) would chase the topologically strained gap out of the 3′-end of the nucleoprotein filament, therefore removing topological roadblocks to extensive DNA strand exchange.

Removing the motoring requirement in the facilitated DNA-rotation model appears to be possible without eliminating the advantage of ATP hydrolysis on strand exchange (the hypothesized model is shown in Fig. 5 ▶). In this revised model, cycles of nucleotide exchange and ATP hydrolysis trigger cycles of conformational change in loop L2 and hence binding and transient release of DNA substrates. Such shake-and-bake cycles are based on the conserved cationic bridge in the ATPase centres of RadA and RecA and on the generally accepted notion that the ATP-hydrolysis cycle controls the transition of the recombinase filament between high- and low-­affinity states for DNA. Homologous pairing between filament-initiating ssDNA and its homologous dsDNA appear to be much faster than the separation of the outgoing ssDNA (Bazemore, Folta-Stogniew *et al.*, 1997 ▶; Bazemore, Takahashi *et al.*, 1997 ▶), especially in AT-rich regions (Folta-Stogniew *et al.*, 2004 ▶). As a result, pairing could happen intermittently between long DNA substrates. The resulting intervening dsDNA gap between paired regions (Fig. 5 ▶ *a*) would be topologically restrained (underwound around the recombinase filament) from pairing despite its sequence homology with the filament-initiating ssDNA. Before strand exchange can proceed to completion, such a roadblock has to be removed (Shan *et al.*, 1996 ▶; Cox, 2003 ▶; Rice *et al.*, 2001 ▶). ATP hydrolysis-triggered conformational change in L2 and transient release of DNA at the gap’s immediate 3′-flank (relative to the polarity of the initiating ssDNA) would be advantageous to this requirement (Fig. 5 ▶ *b*). As the released dsDNA gains temporary freedom, it could unwind around the recombinase filament in synchronization with proper winding of the dsDNA in the 5′-end of the gap (Fig. 5 ▶ *c*). It is worth noting that such rearrangement does not change the overall topology of the system, thus requiring neither motor function (for active rotation) nor the dissociation of recombinase subunits (for passive rotation around a single bond in the uncoated ssDNA). As a result, pairing is enabled in the original gap region, which now becomes properly wound (Fig. 5 ▶ *d*), and the resulting underwound dsDNA gap is displaced to the 3′-flank of the original one. Repetition of such displacement cycles would chase the gap out of the 3′-end of the entire filament, hence removing the topological roadblock to strand exchange (Fig. 5 ▶ *e*). The directionality of gap displacement and the outcome of this revised model is essentially identical to those of the original facilitated DNA-rotation model (see Fig. 5 ▶ of Cox, 2003 ▶), except that facilitated rotation of dsDNA and synchronized ATP hydrolysis in adjacent recombinase monomers are no longer required. Since released dsDNA favours a shorter pitched B-form, the revised model appears to be compatible with the fact that recombinase filaments have shorter pitches in the absence of ATP or as a result of ATP hydrolysis (DiCapua *et al.*, 1990 ▶; Yu *et al.*, 2001 ▶; Story *et al.*, 1992 ▶). The accepted 5′-to-3′ direction of RecA disassembly (Shan *et al.*, 1997 ▶; Cox *et al.*, 2005 ▶) suggests that affinity for DNA at the 5′-end is lowered the most by ATP hydrolysis. In the interior of the filament, an intervening gap would create an internal pseudo 5′-end at the gap’s 3′-flank. Therefore, the 5′-to-3′ direction of gap displacement as proposed should be the preferred directionality consistent with that of strand exchange (Gupta *et al.*, 1998 ▶). Even in the absence of a preferred direction of transient DNA release, the faster 5′-to-3′ filament-growth rate of RecA (Galletto *et al.*, 2006 ▶) would still dictate the direction of gap displacement. Although the directionality of Rad51-promoted DNA strand exchange is an issue of debate, it is easier to comprehend a conserved polarity (Gupta *et al.*, 1998 ▶). Since filament growth is always faster than filament disassembly, as reviewed for RecA (Roca & Cox, 1997 ▶) and recently observed for Rad51 (van der Heijden *et al.*, 2007 ▶; Mine *et al.*, 2007 ▶), it is likely that the trailing 5′-end of the gap keeps pace with its leading 3′-end. RecA appears to hydrolyze ATP in synchronized unidirectional waves which traverse ∼120 subunits per minute on dsDNA (Shan *et al.*, 1997 ▶; Cox *et al.*, 2005 ▶). If gap displacement is to follow such a wave, all gaps would be cleared within 50 min for a 6 kb dsDNA substrate. This rate estimate of gap clearance is consistent with strand-exchange experiments using ϕX174-derived DNA substrates as exemplified in a comparative study on RecA and Rad51 (Rice *et al.*, 2001 ▶). Unlike RecA, eukaryal and archaeal homologues generally lack cooperativity and do not appear to hydrolyze ATP in a synchronized fashion. The likely sporadic nature of ATP hydrolysis by such homologues would lead to much slower gap clearance consistent with their slower rate of promoting strand exchange between ϕX174-derived DNA substrates (Rice *et al.*, 2001 ▶). The main question remains whether this model can be reconciled with the fact that DNA strand exchange takes place in the presence of nonhydrolyzable ATP analogues. Although RecA-like proteins bind DNA, they do not appear to bind short ssDNA tight enough. It is generally accepted that oligonucleotides shorter than 30 nucleotides do not fully activate the ATPase activity of RecA (Brenner *et al.*, 1987 ▶; Bianco & Weinstock, 1996 ▶), which can be attributed to length-dependent affinity between RecA and ssDNA (Singleton *et al.*, 2007 ▶). Similar length-dependence may also exist for dsDNA binding. As a consequence of thermal fluctuation, short stretches of dsDNA can be temporarily released to enable gap displacement. ATP hydrolysis may simply magnify the advantage of transient release of DNA for removing topological roadblocks. The original facilitated DNA-rotation model could explain how coupled ATP hydrolysis enables extensive DNA strand exchange. By assuming a common 5′-to-3′ directionality, our revised model could also explain extensive DNA strand exchange in the absence of ATP hydrolysis or in the presence of uncoupled ATP hydrolysis.

## Supplementary Material

PDB reference: RadA, 3etl, r3etlsf

PDB reference: 3ew9, r3ew9sf

PDB reference: 3ewa, r3ewasf

## Figures and Tables

**Table 1:** Data-collection and refinement statistics Values in parentheses are for the highest resolution shell.

PDB code	3etl	3ew9	3ewa
Monovalent salt	0.1 *M* NH_4_Cl	0.4 *M* KCl	0.4 *M* NH_4_Cl
Data collection			
Space group	*P*6_1_	*P*6_1_	*P*6_1_
Unit-cell parameters			
*a* = *b* (Å)	81.1	81.2	81.1
*c* (Å)	108.5	107.1	107.2
α = β (°)	90	90	90
γ (°)	120	120	120
Resolution (Å)	2.4 (2.5–2.4)	2.4 (2.5–2.4)	2.0 (2.1–2.0)
*R*_merge_	0.069 (0.347)	0.057 (0.379)	0.061 (0.448)
*I*/σ(*I*)	17.1 (4.8)	15.8 (3.0)	17.5 (3.5)
Completeness (%)	96.8 (72.0)	94.6 (83.2)	97.6 (91.6)
Unique reflections	15276 (1033)	15225 (1237)	26987 (3375)
Redundancy	9.9 (9.3)	6.8 (6.1)	10.7 (10.6)
Refinement			
Resolution (Å)	20–2.4	20–2.4	20–2.0
No. of reflections	15276	15225	26987
*R*_work_/*R*_free_	0.222/0.260	0.216/0.260	0.223/0.255
No. of atoms	2364	2449	2483
Protein	2323	2406	2405
Ligand/ion	33	35	33
Water	8	8	45
*B* factor (Å^2^)	47.1	47.5	38.5
Protein	47.2	47.6	38.7
Ligand/ion	39.2	42.4	32.5
Water	31.8	30.4	34.8
R.m.s. deviations			
Bond lengths (Å)	0.0068	0.0071	0.0060
Bond angles (°)	1.22	1.24	1.21

## Footnotes

1 Abbreviations: MmRadA, RadA recombinase from *Methanococcus maripaludis*; MvRadA, RadA recombinase from *M. voltae*; EcRecA, RecA recombinase from *Escherichia coli*; ssDNA, single-stranded DNA; dsDNA, double-stranded DNA; hdDNA, heteroduplex DNA; ATP, adenosine 5′-triphosphate; AMPPNP, 5′-adenylyl imidodiphosphate, a nonhydrolyzable analogue of ATP; FAM, fluorescein phosphoamidite.
